# Structural covariance network patterns linked to neuropsychiatric symptoms in biologically defined Alzheimer's disease: Insights from the mild behavioral impairment checklist

**DOI:** 10.1177/13872877251316794

**Published:** 2025-02-16

**Authors:** Marco Michelutti, Daniele Urso, Benedetta Tafuri, Valentina Gnoni, Alessia Giugno, Chiara Zecca, Maria Teresa Dell’Abate, Davide Vilella, Paolo Manganotti, Roberto De Blasi, Salvatore Nigro, Giancarlo Logroscino

**Affiliations:** 1 Center for Neurodegenerative Diseases and the Aging Brain, Department of Clinical Research in Neurology, University of Bari ‘Aldo Moro’, “Pia Fondazione Cardinale G. Panico”, Lecce, Italy; 2 Clinical Unit of Neurology, Department of Medicine, Surgery and Health Sciences, University Hospital of Trieste, University of Trieste, Italy; 3 Department of Neurosciences, King's College London, Institute of Psychiatry, Psychology and Neuroscience, London, UK; 4 Department of Translational Biomedicine and Neuroscience (DiBraiN), University of Bari Aldo Moro, Bari, Italy; 5 Department of Diagnostic Imaging, Pia Fondazione di Culto e Religione “Card. G. Panico”, Italy; 6 Institute of Nanotechnology, National Research Council (CNR-NANOTEC) c/o Campus Ecotekne, Lecce, Italy

**Keywords:** Alzheimer's disease, graph theory, mild behavioral impairment, neuropsychiatric symptoms, structural covariance

## Abstract

**Background:**

The frequent presentation of Alzheimer's disease (AD) with neuropsychiatric symptoms (NPS) in the context of normal or minimally-impaired cognitive function led to the concept of Mild Behavioral Impairment (MBI). While MBI's impact on subsequent cognitive decline is recognized, its association with brain network changes in biologically-defined AD remains unexplored.

**Objective:**

To investigate the correlation of structural covariance networks with MBI-C checklist sub-scores in biologically-defined AD patients.

**Methods:**

We analyzed 33 biologically-defined AD patients, ranging from mild cognitive impairment to early dementia, all characterized as amyloid-positive through cerebrospinal fluid analysis or amyloid positron emission tomography scans. Regional network properties were assessed through graph theory.

**Results:**

Affective dysregulation correlated with decreased segregation and integration in the right inferior frontal gyrus (IFG). Impulse dyscontrol and social inappropriateness correlated positively with centrality and efficiency in the right posterior cingulate cortex (PCC). Global network properties showed a preserved small-world organization.

**Conclusions:**

This study reveals associations between MBI subdomains and structural brain network alterations in biologically-confirmed AD. The IFG's involvement is crucial for mood dysregulation, while the PCC could be involved in compensatory mechanisms for social cognition and impulse control. These findings underscore the significance of biomarker-based neuroimaging for the characterization of NPS across the AD spectrum.

## Introduction

The onset of mild cognitive impairment (MCI) due to Alzheimer's disease (AD) has been seen to be heralded by neuropsychiatric symptoms (NPS) in the absence of cognitive complaints in approximately 30% of the patients.^1^ Furthermore, the presence of NPS has been observed to anticipate the conversion from MCI to dementia in approximately 40% of the patients.^1^ The presence of a wide spectrum of late-onset persisting NPS in pre-dementia stages has been formalized as mild behavioral impairment (MBI).^2,3^ Despite initially studied in the prodromal stage of behavioral variant-frontotemporal dementia, MBI has increasingly received attention in the context of AD.^4^ MBI increased the risk of progression to MCI independently from subjective cognitive decline (SCD);^5^ furthermore, MBI was linked to declines in executive function, attention, and episodic memory, regardless of cognitive status.^5^ For these reasons it has been postulated that the two may be complementary constructs.^6^

The MBI-checklist (MBI-C) has been developed according to the ISTAART-MBI criteria.^7^ It is tailored to pre-dementia subjects as opposed to measures used in older studies such as the Neuropyschiatric Inventory (NPI),^2^ which were aimed at detecting NPS in later-stages of cognitive impairment.^8–10^ However, the MBI-C has been also previously used in the context of overt dementia.^11–13^ In fact, the MBI-C revealed a higher prevalence of NPS than the NPI in patients with SCD, MCI, and dementia.^13^

For all these reasons, in our current study we investigate the patterns of structural covariance networks associated with neuropsychiatric impairment assessed through the MBI-C rather than the NPI. We perform this analysis in a biologically-defined AD cohort across the spectrum from MCI to early dementia, by combining structural covariance and a graph theory approach.^14–17^

In the past years, neuroimaging correlates of MBI have been investigated in several normal and pathological conditions.^18–20^ Regional gray and white matter changes were assessed in pre-dementia MBI state in NC,^21,22^ SCD,^23^ MCI^23,24^ as well as in subjects defined as MBI-impulse dyscontrol-positive considering a mixed cohort of clinically-defined NC, MCI, and AD-dementia patients.^18^ A cohesive account for the findings of regional atrophy across these studies is still lacking. However, frontal and temporal atrophy have been indicated both in cognitively unimpaired^22^ and in a SCD/MCI cohort of MBI subjects.^24^ A more widespread pattern, also involving inferior parietal cortex, has been documented to be present in MBI patients whose alterations were reported for more than one neuropsychiatric domain.^24^ Functional connectivity has also been explored by means of resting-state functional magnetic resonance imaging (rs-fMRI) in MBI-positive, dementia-free subjects ranging from normal cognition to MCI.^19,20^

Despite recent progress in understanding brain changes associated with MBI, to date no study has used the inter-regional gray matter covariation to investigate the association between MBI and brain network properties in patients with biologically-defined AD. Structural covariance analysis has proved to be a robust approach to investigate the brain network organization in several neurological conditions, including AD.^14,25,26^ Furthermore, an increasing body of evidence supports the notion that alterations in the topological structure of brain networks are linked to cognitive or behavioral impairments.^27–29^ This approach relies on the assumption that morphological properties of interconnected brain regions covary due to shared developmental and maturational influences.^30^ Concerning MBI investigations, to our knowledge, only one study has investigated structural network changes in a cohort of cognitively normal older adults with and without MBI, observing a reduced segregation in networks including frontal and parietal regions in subjects with MBI.^31^ This prior study, as well as other previous connectivity investigations, considered only clinically defined “dementia-free” subjects.^19,20,31^ However, NPS might precede cognitive impairment in a series of different pathologies such as behavioral variant frontotemporal dementia and Parkinson's disease.^32–34^ These symptoms can present at an early stage of such diseases, possibly leading to clinical overlap.^6^ This implies that the neuroimaging correlates of MBI in clinically defined “dementia-free” subjects may be influenced by distinct underlying neurodegenerative mechanisms.^35,36^ For this reason, previous attempts at the identification of a neuroimaging signature underlying the early onset of NPS in normal cognition and MCI subjects might have been confounded by sub-optimal inclusion criteria based on a clinical rather than a biological diagnosis.

Considering the previously reported studies,^19,20,31^ we expected changes in the fronto-parietal information exchange to correlate with MBI-C sub-scores. To examine this hypothesis, we utilized the following local graph metrics: (i) a centrality measure, specifically the degree centrality, (ii) a segregation measure, including the clustering coefficient, and (iii) an integration measure, specifically the nodal efficiency.

## Methods

### Participants

All participants were directed to the Center for Neurodegenerative Diseases and the Aging Brain of the University of Bari Aldo Moro at Pia Fondazione “Card. G. Panico”. All the subjects were referred to the center for cognitive complaints. These individuals underwent an extensive assessment, which encompassed a clinical history review, neurological examination, and neuropsychological testing. The patients were selected from 392 subjects who had been administered the MBI-Checklist (MBI-C)^7^ from January 2021 to June 2023.

Other inclusion criteria for the study were: a diagnosis of AD according to the research framework NIA-AA 2018 criteria^4^ and availability of 3T structural MRI. Amyloid positivity was confirmed by CSF analysis or amyloid-PET-positivity.^4^ Exclusion criteria for the study were the presence of: moderate-advanced stage cognitive impairment (CDR > 1);^37^ atypical phenotype of AD, i.e., logopenic variant primary progressive aphasia (lvPPA),^38^ dementias other than AD (i.e., frontotemporal dementia) or a primary diagnosis of a movement disorder (i.e., Parkinson's disease); other neurological or comorbid primary psychiatric disease; clinical or neuroimaging indications of focal lesions, as well as the presence of other inflammatory, infectious, or vascular conditions. Three-hundred and thirty-five patients were not included because they were not diagnosed as AD patients according to the NIA-AA 2018 criteria. Thirty-seven patients met the inclusion criteria. Two patients were excluded from the study because their stage of cognitive impairment was moderate (CDR = 2). Finally, other 2 patients were excluded from the study due to atypical phenotype (lvPPA). The presence of brain amyloidosis was deemed positive by means of amyloid PET in 6 patients; 27 patients received the diagnosis of AD according to CSF biomarkers values. The patients underwent lumbar puncture for the dosage of CSF biomarkers of neurodegeneration (Amyloid-β 1-42 [Aβ_42_], total Tau and phospho-Tau181) according to standard procedures. The levels of CSF Aβ_42_, total Tau (t-Tau), and p-Tau181 were quantified using a chemiluminescent immunoassay CLEIA (Lumipulse G β-amyloid 1–42, Lumipulse G Total Tau, Lumipulse G pTau181) conducted on a fully automated platform (Lumipulse G600II) by Fujirebio Europe N.V. in Gent, Belgium. All the assays were performed according to manufacturer's protocols. For the interpretation of the cerebrospinal biomarker results, the following cut-off values were considered: Aβ_42 _< 599  pg/mL, t-Tau > 342  pg/mL, p-Tau181 > 57  pg/mL. These were provided directly by the manufacturer.

All the patients had a positive amyloid status (A+). Only two patients had a negative p-Tau181 marker (A + T-N+). However, these could be classified as having a CSF profile compatible with AD since they had a positive t-Tau/Aβ_42_ ratio.^39^

Each participant provided written informed consent. The study was conducted according to the guidelines of the Declaration of Helsinki and approved by the Institutional Review Board of ASL Lecce (verbale n. 6, 25 July 2017).

### MBI-C assessment

Ismail et al.^7^ and Kang et al.^40^ developed the MBI-C, a questionnaire which was administered to the caregivers of the patients. The questionnaire consisted of 34 items grouped into five domains: Drive/Motivation, Mood/Anxiety, Impulse dyscontrol, Social inappropriateness, and Perception/Thought. Each item presented a binary choice (‘yes’ or ‘no’) question, followed by a severity rating scale ranging from 1 (mild) to 3 (severe). To meet the criteria, the symptoms needed to persist for at least six months and represent a significant deviation from the baseline. Additionally, we utilized an adapted version of the questionnaire specifically for the Italian population. This was translated from English in collaboration with the authors of the original MBI-C.^41^

Based on the distribution of scores in our sample, the patients were categorized as either having a MBI score higher or lower than the median value, which was equal to or greater than 16.

### Neuropsychological examination and medication status

All individuals underwent an extensive neuropsychological assessment designed to evaluate five cognitive domains (attention, executive functioning, language, memory visuospatial ability, and social cognition). The neuropsychological evaluation included the following tests: Clinical Dementia Rating (CDR),^37^ Mini-Mental State Examination (MMSE),^42^ the Rey Auditory Verbal Learning Test (RAVLT)^43^ and the Delayed Recall of “Rey Complex Figure”.^44^ Frontal Assessment Battery (FAB),^45^ Digit Span Backwards and Forwards,^46^ Phonemic and Semantic Verbal Fluencies,^47^ Boston Naming Test (short-version),^48^ incomplete Letters sub-test of VOSP battery,^49^ Design Copy Test,^50^ Rey-Osterrieth complex figure copy,^51^ Clock Drawing Test,^52^ Trail-Making Test;^53^ Symbol Digit Modality Test;^54^ Stroop Test;^55^ Social Cognition subtest of the Edinburgh Cognitive and Behavioural ALS Screen;^56^ Basic and Instrumental Activities of Daily Living and the Neuropsychiatric Inventory.^57^ A neuropsychologist administered and scored each test, and the raw scores were converted to z-scores based on appropriate normed data. Subsequently, domain specific average z-scores were calculated. Subjects were classified as having MCI according to clinical criteria.^58^ These requirements were as follows: (1) performance that fell more than 1.5 standard deviations below the standardized mean on a minimum of 2 tests within or across cognitive domains; (2) a self-reported or caregiver-noted subjective complaint of cognitive decline; (3) the absence of a notable decline in daily living activities; and (4) the absence of a dementia diagnosis. Subjects were classified as having dementia if Activities of Daily Living (ADL) and Instrumental Activity of Daily Living (IADL)^57^ were found to be compromised in respect to baseline.

The presence of medication that could have had an effect on MBI-C subscores was assessed. In details, it was reported whether the patients were taking antidepressants, antipsychotics, hypnotics, or anxiolytics at the time of the assessment. Furthermore, total anticholinergic medication burden for each patient was calculated using the Anticholinergic Burden (ACB) score.^59,60^ The ACB scale categorizes medications by assigning scores ranging from 0 to 3, which are determined by their interaction with muscarinic receptors and their impact on cognitive function. A score of 1 suggests an affinity for muscarinic receptors but no significant documented cognitive effects. Scores of 2 signify mild cognitive effects, while scores of 3 indicate more pronounced and established adverse cognitive effects. Finally, it was reported whether the patients were receiving anti-cholinesterase inhibitors (AChE-I) or memantine at the time of the assessment.

### MRI acquisition and preprocessing

The structural images were obtained using a 3T scanner, specifically the Philips Ingenia 3.0T, employing a Fast-Field Echo (FFE) T1-weighted sequence. The imaging parameters were set as follows: a repetition time of 8.2 ms, an echo time of 3.8 ms, a field of view measuring 256 × 256 mm^2^, 200 slices, a flip angle of 8 degrees, and voxels with an isotropic size of 1 mm^3^. All T1-weighted images were initially visually inspected for gross structural alterations and artifacts, and subsequently, preprocessed using voxel-based morphometry implemented in the Computational Anatomy Toolbox (CAT12, http://www.neuro.uni-jena.de/cat). Specifically, the pre-processing pipeline included corrections for bias-field inhomogeneities and segmentation into grey matter, white matter and cerebrospinal fluid. Next, gray matter (GM) images were subjected to non-linear coregistration by means of the Diffeomorphic Anatomical Registration Through Exponentiated Lie Algebra (DARTEL) algorithm, and modulated to guarantee that relative volumes were preserved following the spatial normalization procedure. Subsequently, all modulated gray matter images underwent a smoothing process with an isotropic Gaussian kernel having a full-width-half-maximum (FWHM) of 8 mm.

### Network construction

Individual structural covariance networks were constructed using the method proposed by Kong and colleagues.^61–63^ Briefly, for each subject, the automated anatomical labeling (AAL) atlas was used to parcellate the GM into 90 regions of interest (*network nodes*).^64^ Next, the GM values were extracted within each brain region and the probability density function (PDF) of these values were estimated using kernel density estimation (KDE).^62^ Then, the structural connectivity value (*network edges)* between each pair of regions was quantified using the symmetric Kullback–Leibler (KL) divergence-based similarity.^61^ In particular, the KL divergence, defined as the statistical similarity of PDFs between brain regions, was converted to a similarity measurement using the following equation:

$$KLS(\;p,\;q)\;=\;e^{-KL(p,q)}$$

where p and q are the two PDFs. In this way, we generate a 90 × 90 connectivity matrix for each subject, where each row and column represent a brain region, and each element represents the similarity of GM value distributions between each pair of regions. The range of possible KLS values is 0 to 1, where 1 represents an identical distribution for the two regions.

### Graph theory analysis

The estimation of global and local network characteristics was conducted using the Graph Theoretical Network Analysis (GRETNA) package (available at www.nitrc.org/projects/gretna/)63.

To assess the global topological organization of the covariance structural networks, small world measures were employed. In this regard, we computed the normalized clustering coefficient (γ = Cp _real_/Cp_random_) and the normalized characteristic path length (λ = Lp_real_/Lp_random_). Subsequently, the small-world index was determined as the ratio of the normalized clustering coefficient to the normalized path length (σ = γ/λ). It is important to note that Cp_real_ and Lp_real_ represent the clustering and characteristic path length of the real network, respectively, while Cp_random_ and Lp_random_ denote the mean clustering coefficient and shortest path length of 1000 matched random networks that preserve the same number of nodes, edges, and degree distribution as the real network. A network can be considered a small-world network if it meets the following criterion: a small-world index σ = λ/γ > 1.1.^65^ Compared to a random network, a small-world network exhibits a higher clustering coefficient and a characteristic path length similar to that of a random network.

Regional network properties were assessed through the degree centrality, clustering coefficient, path length, and efficiency.^65^ Degree centrality quantifies the relative importance of a node within a network, while the clustering coefficient reflects a node's ability to communicate with other nodes it shares direct connections with (segregation ability). Nodal efficiency and characteristic path length evaluate the ability of information propagation between a node and the remaining nodes in the network (integration ability). A node with high nodal efficiency or a low path length is characterized by a strong capability for information transmission to other nodes. More detailed explanations and formulas for these global and local metrics can be found in previous methodological reviews.^66–68^

Given that graph measures are dependent on the underlying graph's density, we applied thresholding to the intra-individual structural covariance networks within a density range of d = 0.10–0.40, with an interval of 0.02. This approach is commonly used to eliminate noisy or spurious connections while retaining the most robust structural edges. The chosen density range allows for an accurate estimation of small-world network properties and minimizes the number of spurious connections in each network.^69–71^ Subsequently, network parameters were calculated for each network at each density. To provide a scalar metric that does not depend on specific threshold selection, GRETNA computed the area under the curve (AUC) for each network measure, representing the integral over the density range.^72,73^

### Statistical analysis

The Shapiro-Wilk test was conducted on various categories of data, including demographic, neuroimaging, and neuropsychological variables (e.g., age, total intracranial volume, cognitive performance), as well as graph measures, in order to assess the normality of data distribution.

Next, variables with a normal distribution were compared between patients with an MBI-C score ≥ 16 (the median MBI-C score in our sample) and patients with a MBI-C score < 16 using pair-wise t-tests. For variables that did not follow a normal distribution, group comparisons were made using the Wilcoxon-Mann-Whitney test. The critical statistical threshold was set to p < 0.05. A false discovery rate (FDR) correction procedure was employed to correct for multiple comparisons in the clinical data. Spearman's correlations were performed between the MBI-C total and subscores and neuropsychological tests, CSF biomarker values and demographic features.

The relationships between brain network metrics and clinical data (MBI-C total score) were tested using partial correlation, accounting for covariates such as sex, age, disease duration, and total MMSE score. The correlations were considered statistically significant when p-value was lower than 0.05 after FDR correction.

## Results

### Demographic and clinical features

Thirty-three AD patients (males = 10; females = 23; mean age = 69.75 ± 6.88) were included in the study.

Sixteen of the patients were diagnosed as affected by early-stage dementia (CDR 1.0, males = 6, mean age = 69 ± 4.60); the other 17 patients were diagnosed as MCI (CDR 0.5, males = 4, mean age =70.47 ± 8.58).

The mean total MBI-C scores were 12.41 ± 9.57 and 19.81 ± 14.19 for the MCI and early dementia groups, respectively. The mean subscores were 3.52 ± 3.59 and 5 ± 3.19 for the MCI and AD groups respectively in the MBI-drive/motivation subdomain; 2.82 ± 3.46 (MCI) 4.12 ± 3.46 (AD) in the MBI-mood/anxiety subdomain; 4.29 ± 3.77 (MCI) 7.62 ± 7.01 (AD) in the MBI-impulse dyscontrol subdomain; 1 ± 1.11 (MCI) and 1.81 ± 2.34 (AD) in the MBI-social inappropriateness subdomain; 0.76 ± 1.71 (MCI) and 1.25 ± 1.91 (AD) for the MBI-perception/thought subdomain.

The MCI-C total score and single-domain subscores did not differ significantly between the MCI and early dementia groups.

No differences were found in age, sex and years of education between AD patients with MBI-C score higher and lower than the median score. Concerning clinical data, patients with MBI-C score higher than 16 had only significantly higher disease duration (p = 0.002) than those with lower MBI-C score, while other clinical variables were not significantly different between the two groups. No significant difference was observed in the frequency of administration of antidepressants, antipsychotics, hypnotics, anxiolytics as well as of AChE-I and memantine between the two groups. No correlation was significant between MBI-C total and subscores and any of the clinico-instrumental parameters taken into consideration (neuropsychological tests, CSF biomarkers, demographic features).

The demographic and clinical features of our cohort are described in Table 1 and Supplemental Table 1.

**Table 1.** Demographic and clinical features.

	Whole group		MBI-C < 16		MBI-C ≥ 16		
	Mean	Std. Deviation	Mean	Std. Deviation	Mean	Std. Deviation	*p* (non parametric)
Age	66.372	15.764	67.705	7.880	71.938	5.000	0.058
Disease Duration	2.742	1.742	1.853	0.702	3.688	2.024	**0**.**002**
Years of Education	9.879	5.017	10.824	5.294	8.875	4.660	0.309
Aβ_42_-amyloid (pg/mL)	533.259	145.237	531.929	87.523	534.692	193.391	1.000
t-Tau (pg/mL)	829.852	336.918	849.071	299.242	809.154	384.764	0.616
p-Tau181(pg/mL)	132.367	58.706	133.621	52.056	131.015	67.289	0.557
CDR: total	0.667	0.323	0.618	0.376	0.719	0.256	0.423
CDR: sum of boxes	3.000	2.207	2.875	2.760	3.125	1.555	0.491
MMSE	19.129	5.419	19.059	4.657	20.063	5.397	0.943
Cognitive Reserve Index	96.741	19.823	95.867	19.497	97.833	21.040	0.860
FAB	10.857	3.336	10.615	3.097	11.250	3.882	0.465
Memory	−2.148	0.949	−1.994	1.067	−2.313	0.808	0.204
Executive Functions	−1.685	1.261	−2.008	1.503	−1.341	0.858	0.631
Attention	−2.102	4.942	−1.493	1.829	−2.748	6.905	0.510
Language	−1.445	1.177	−1.595	1.218	−1.286	1.149	0.326
Visuo-spatial processing	−2.015	2.110	−2.488	2.549	−1.511	1.431	0.432
Social Cognition	−1.279	1.319	−1.476	1.383	−1.110	1.289	0.790
ADL	5.727	0.911	5.647	1.222	5.813	0.403	0.074
IADL	6.455	1.804	6.941	1.519	5.938	1.982	0.191
Anticholinergic burden	0.970	1.015	0.941	0.899	1.000	1.155	0.901
	**Frequency (%)**		**Frequency (%)**		**Frequency (%)**		***p***
Sex: males (%)	10 (30.3%)		3 (9%)		7 (21.2%)		0.204
Antidepressant drugs (%)	16 (48.48%)		9 (27.27%)		7 (21.21%)		0.289
Antipsychotic drugs (%)	1 (3.30%)		0		1 (3.30% %)		0.813
Hypnotic drugs (%)	2 (6.06%)		1 (3.30%)		1 (3.30%)		0.944
Anxiolytic drugs (%)	2 (6.06%)		2 (6.06%)		0		0.502
AChE-I (%)	8 (24.24%)		4 (12.12%)		4 (12.12%)		0.726
Memantine (%)	5 (15.15%)		1 (3.30%)		4 (12.12%)		0.509

Independent sample *t* test and Wilcoxon-Mann-Whitney test were used to compare values between the two groups. Significant *p* values are highlighted in bold. Abbreviations: MBI-C: Mild Behavioral Impairment Checklist; CDR: Clinical Dementia Rating; FTD: Frontotemporal dementia scale; UPDRS: Unified Parkinson's Disease Rating Scale; MMSE: Mini-mental State Examination; FAB: Frontal Assessment Battery; ADL: Activity of Daily Living; IADL: Instrumental Activity of Daily Living; AChE-I: anti-cholinesterase inhibitors; t-Tau: total Tau; p-Tau181: phosphorylated Tau 181.

MBI-C total and sub-scores were not significantly different between patients with CDR 1.0 and 0.5. There were no correlations between the MBI-C total score, its subdomains (Decreased Motivation, Affective Dysregulation, Impulse Dyscontrol, Social Inappropriateness, and Perception/Thought), and the other clinical variables, including MMSE, CDR-total score, CDR-sum of boxes, cognitive domain z-scores, FAB, ADL, IADL, anticholinergic burden,^59,60^ as well as CSF values (Aβ_42_, t-Tau, p-tau181).

### Global network properties

The structural covariance network of the patients demonstrated small-world network architecture (1.2 < σ < 2.7) over the preselected density range. No significant correlation was found between global network parameters and MBI-C total score as well with MBI-C subscores.

### Regional network properties

**Figure 1.**
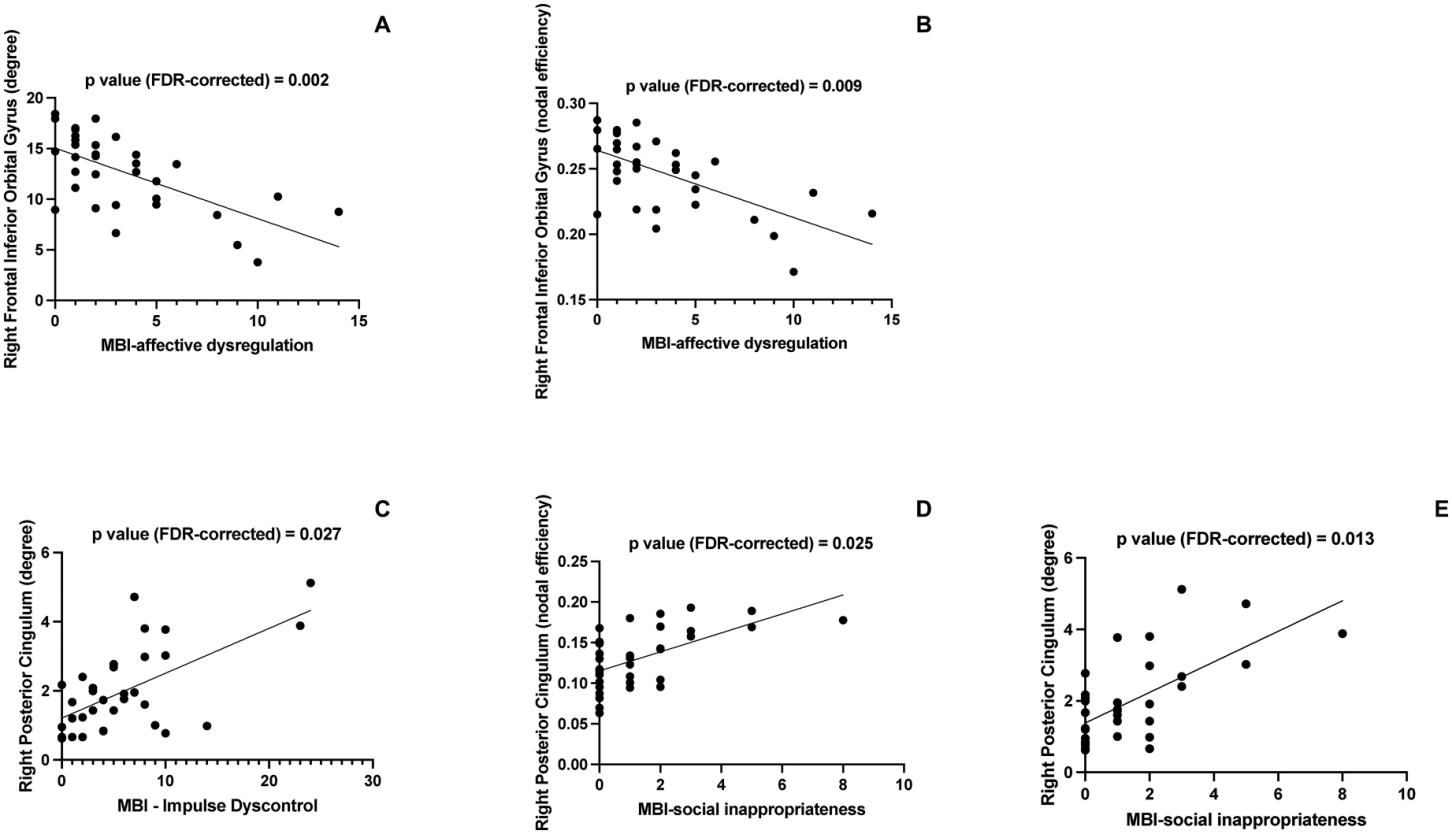
Regional network correlates of MBI-C subscores in Alzheimer's disease. (A) Correlation between MBI-C affective dysregulation score and the degree of the right frontal inferior orbital cortex. (B) Correlation between MBI-C affective dysregulation score and the efficiency of the right frontal inferior orbital cortex. (C) Correlation between MBI-C social inappropriateness score and the degree of the right posterior cingulate cortex. (D) Correlation between MBI-C social inappropriateness score and the efficiency of the right posterior cingulate cortex. (E) Correlation between MBI-C impulse dyscontrol score and the degree of the right posterior cingulate cortex.

As shown in Table 2 and Figure 1, the MBI-C affective dysregulation sub-score was found to be inversely correlated with both the degree (R = -0.69, FDR-corrected p = 0.0026) and the nodal efficiency (R = -0.72; FDR-corrected p = 0.009) of the right inferior frontal cortex. The MBI-C social inappropriateness sub-score was found to be positively correlated with both the degree (R = 0.64; FDR-corrected p = 0.013) and the nodal efficiency (R = 0.63 ; FDR-corrected p = 0.025) of the right posterior cingulum. A positive correlation was also found between the MBI-C Impulse Dyscontrol sub-score and the degree of the right posterior cingulum (R = 0.62; FDR-corrected p = 0.027). Of note, no significant correlations were found between local network parameters and MBI-C total score, as well as with other clinic-instrumental variables such as MMSE, and cognitive domain scores.

Frontal_Inf_orb_R: inferior orbital part of the right frontal cortex; Cingulum_post_R_ right posterior cingulate cortex.

**Table 2.** Correlation between MBI-C scores and local structural connectivity parameters.

			MBI-C: total score	MBI-C: decreased motivation	MBI-C: affective dysregulation	MBI-C: impulse dyscontrol	MBI-C: social inappropriateness	MBI-C: perception/thought
Frontal-inferior Orbital-Right								
	Degree	Correlation Coefficient	−0.390	−0.400	−0.690	−0.110	−0.110	0.006
		p	0.030	0.030	0.000	0.540	0.550	0.970
		p (FDR-corrected)	No	No	**0** **.** **002**	No	No	No
	Nodal Efficiency	Correlation Coefficient	−0.420	−0.430	−0.720	−0.090	−0.11	0.050
		p	0.030	0.020	0.000	0.630	0.540	0.780
		p (FDR-corrected)	No	No	**0**.**009**	No	No	No
Cingulum Posterior-Right								
	Degree	Correlation Coefficient	0.556	0.089	0.250	0.620	0.640	0.480
		p	0.001	0.640	0.190	0.000	0.000	0.007
		p (FDR-corrected)	No	No	No	**0**.**027**	**0**.**013**	No
	Nodal Efficiency	Correlation Coefficient	0.510	0.040	0.300	0.560	0.630	0.410
		p	0.004	0.840	0.100	0.001	0.00028	0.030
		p (FDR-corrected)	No	No	No	No	**0**.**025**	No

Partial correlation was calculated using sex, age, MMSE score and disease duration. Values expressed as correlation coefficient R; p: the corrected p value is highlighted in bold when significant after FDR correction.

## Discussion

In this study, we combined structural covariance and graph-theory analyses to investigate the associations between neuropsychiatric impairment identified by MBI and the topological properties of the structural brain networks in patients with a biologically-defined diagnosis of AD. Our investigation unveiled significant correlations between MBI sub-scores and network properties in specific brain regions associated with behavioral regulation. In particular, MBI-affective dysregulation scores were inversely correlated with the degree and the nodal efficiency of the orbital part of right the inferior frontal gyrus (IFG). Positive correlations, instead, were found between the regional network properties of the right posterior cingulum cortex (PCC) and MBI-impulse dyscontrol subscores (degree) and MBI-social inappropriateness scores (degree and nodal efficiency). The clinical, neuropsychological and instrumental (i.e., CSF biomarkers) features of the patients were not correlated with the MBI status. This supports the hypothesis that MBI is a construct independent from the cognitive status.

Our findings provide new insights into our understanding of the associations between brain network properties and MBI across the AD spectrum. In particular, a globally preserved small-world organization of structural brain networks (σ > 1) mirrors previous findings observed in cognitively normal subjects with MBI^31^ and in previous studies comparing early AD patients to controls.^74–76^ The latter demonstrated that, despite alterations of individual graph metrics and a tendency to randomization were documented by structural^74,75^ and functional^76,77^ covariance network analyses, such networks retained a general small-world organization,^74–77^ as observed in our study. This provides additional evidence in favor of the theory positing that the small-world topology serves as an especially resilient principle governing the organization of networks in the human system.^78–82^

At the local level, the presence of affective dysregulation was associated with a decreased segregation and integration ability of the IFG in the information processing across brain networks. The IFG has been shown to be involved in depression by means of functional^83–89^ and structural connectivity studies.^89–92^ In particular, alterations of the right IFG structural connectivity were revealed by diffusion tensor imaging in late-life depression in subjects both cognitively normal^89^ and suffering from memory deficits.^91,92^ Concerning previous studies on MBI, Shu et al. found that betweenness centrality, a local connectivity indicator that measure the influence of a node on the information flow, was lower in frontal brain regions encompassing the right IFG when patients with MBI were compared to controls.^31^ In line with this study, our findings highlight the key role of the IFG in processing and transmitting affective information, providing further evidence that a reduced centrality in this brain region may be associated with affective dysregulation.

In addition to changes in the IFG-including fronto-parietal control network,^20^ further alterations in affective regulation measured through the MBI-C have been previously linked to local alterations, such as atrophy^24,93^ and tau deposition,^21,94^ located in temporal and parietal regions. The absence from our findings of topological properties that significantly correlated with MBI in such regions could be mainly due to the methodological differences between our study and those aimed at detecting local alterations. In fact, these are not necessarily reflected by changes in the topology of structural covariance networks.

Concerning the impulse-dyscontrol and social inappropriateness domains, positive associations were found between the MBI subscores and the centrality and integration ability of the right PCC. The PCC is considered a crucial hub for the posterior default mode network regulation^95,96^ and is known to be heavily involved in AD progression.^97–100^ Most importantly, both structural and functional alterations of the PCC have been specifically attributed to the presence of NPS in AD. In particular, lower measures of microstructural integrity of the cingulum bundle have been associated with irritability and agitation in clinically-defined MCI and early AD.^101^ In a cohort of preclinical (amyloid and tau-positive) AD patients the presence of irritability and lability at baseline, among other NPS, predicted the subsequent development of hypometabolism in PCC.^102^ Although our findings might appear in contrast with these investigations showing a reduced structural and functional integrity of the PCC in AD patients; however, recent studies also reported an increased functional connectivity between the PCC and the lateral orbitofrontal cortex in depression.^87^ Given the role of PCC in the default mode network, it has been postulated that enhanced orbitofrontal-PCC connectivity with PCC hyperactivity^31^ might be linked to sad ruminating thoughts in depression.^31,87^ Local measures of increased metabolism in the PCC at baseline were also documented in the presence of irritability and lability.^102^ Moreover, a higher efficiency in the information processing of the precuneus, a region closely linked with the PCC from both an anatomical and functional perspective,^103,104^ have been found by Shu et al. in clinically-defined normal cognition MBI subjects as opposed to healthy controls.^31^ Overall, our findings highlight the involvement of the PCC in impulse dyscontrol and social inappropriateness. However, due to the conflicting findings reported in the literature, including alterations of local properties in regions different than the parietal hubs^18^ further studies are needed to understand the neurophysiological mechanisms underlying the positive association between network properties and MBI scores observed in our study.

Of note, while widespread alterations in fronto-parietal regions were found in previous studies investigating connectivity changes related to MBI correlates,^19,20,31,105^ we found that the MBI subscores of affectivity, impulse dyscontrol and social inappropriateness were associated with local network properties of specific brain regions such as the IFG and PCC. This difference could be partially attributed to the methodological differences between our graph analysis approach and the seed-based connectivity analyses performed in these studies.^19,20^ On the other hand, a correlation between domain-specific MBI-C subscores and connectivity measures was not performed in the graph-theory study of Shu et al.^31^ This impedes the accurate localization of their reported connectivity changes. In fact, MBI-C total score accounts for a global effect caused by NPS that are not necessarily related to each other from the pathogenetical viewpoint. In this way, MBI-C total score could fail to effectively correlate with neuroimaging patterns specific to distinct neuropsychiatric sub-domains. Finally, the fact that these studies did not use a biological definition of AD in their inclusion criteria could have played a role.

No correlation was found between regional graph metrics and clinical cognitive scores such as MMSE and cognitive-domain scores. We speculate that this could be due to different reasons. Firstly, the presence of NPS as identified by MBI has been indicated to be independent from cognitive status.^5^ This is also reinforced by the fact we did not find any significant difference between the MBI-C total and subscores of the MCI and the early dementia groups. Secondly, the integration of structural with functional networks of brain regions poses a significant challenge in connectivity research. An apparently counterintuitive example of the complexity of such relations was put forward by He et al.^75^ In their study, they showed that a network with lower structural connectivity due to atrophy can support higher functional connectivity by means of compensatory activity from secondary sites. This can result in apparent discrepancies between structural and functional connectivity measures as well as with clinical cognitive scores. In fact, cognitive performance might not be linearly correlated with connectivity parameters due to compensatory rerouting of neural connections.

In comparison to individual-based structural and functional connectivity, potentially individualized morphometric brain connectomes are particularly advantageous for studies focused on identifying biomarkers related to development, aging, and brain disorders. This is due to several reasons: (i) individualized morphometric brain connectomes have consistently demonstrated high robustness, reproducibility, and reliability; (ii) they represent one of the simplest, fastest, and most cost-effective methods for generating MRI-based brain networks, making them highly suitable for large-scale, multicenter collaborative studies; and (iii) structural MRI, compared to diffusion and functional MRI, offers distinct benefits such as widespread accessibility, high signal-to-noise ratio, high spatial resolution, and reduced susceptibility to artifacts like head motion.^106^ For these reasons, despite the absence of functional connectivity data in our study, the structural covariance data we have collected remain highly valuable.

This is the first study that investigated the topological abnormalities in structural networks driven by impairment in the MBI subdomains in a sample of biologically-defined AD patients. The unique strength of our study lies in the utilization of biologically-defined AD patients, setting it apart from previous research that primarily relied on clinically defined subjects with normal cognition^31^ and MCI.^18–20^ In contrast to these previous studies, our research incorporates information on the underlying biological etiology, which enhances our ability to establish the specificity of our findings to AD as opposed to other potential pathologies. Moreover, our study distinguishes itself from prior research endeavors that employed metrics other than MBI-C for evaluating the burden of NPS. MBI-C scores have been proved to be a reliable tool not only to predict the risk of a further cognitive impairment, but also to assess the current psychological and behavioral changes of MCI and early-AD patients.^12^ Despite being conceived for pre-dementia patients, the MBI-C was more sensitive in revealing the presence of NPS not only in SCD and MCI but also in patients already affected by dementia.^13^ This difference was partially attributed to a better characterization of apathy and social inappropriateness in the MBI-C than in the NPI.^13^ Furthermore, the MBI-C has been previously demonstrated to detect moderate to severe AD dementia with a higher rate than the NPI.^11^ Therefore, we think that MBI-C scores might reflect the neuroimaging alterations underlying NPS not only in MCI patients but also in patients with early dementia due to AD more accurately than NPI scores, thus being reliably representative of potential underlying structural neuroimaging alterations in both the MCI and dementia patients of our cohort. For this reason, we regarded the MBI-C as an equally effective indicator of potential structural connectivity correlates in the brain for early AD patients as it is for MCI patients.

The burden of MBI-C detectable NPS was observed not to have an effect on any of the demographic, clinical and instrumental (CSF biomarkers) taken into consideration, with the exception of disease duration. In fact, this was found to be significantly higher in patients with a higher MBI burden. This is in line with previous reports of increasing severity of NPS with disease progression in AD.^107^

The absence of correlation between MBI-C scores and CSF biomarkers is unexpected and in contrast with previous works that linked MBI-C burden with tau presence demonstrated either by PET or by CSF sampling.^94^ This might be due to differences between the study cohorts, as these findings were documented in cognitively unimpaired subjects.^94^ Further studies are needed to clarify these observations.

It is important to consider some limitations when interpreting our findings. Firstly, we need to acknowledge that our sample had a relatively limited size. Secondly, our study lacked a control sample. This would have been instrumental to confirm that the correlations we describe between clinical phenotype and structural connectivity measures are specific to MBI-involvement rather than to AD-progression. Future studies should explore if these correlations are exclusive to MBI-due-to AD.^21^ Furthermore, the availability of a control sample would have allowed to quantify the deviation from small-worldness to randomization of the structural covariance network. For these reasons, our study can be viewed as a pilot observation. Thirdly, we acknowledge that the categorization of our patients in two groups according to their MBI-C score was not made according to previously used cut-offs.^13^ Instead, we relied on the median MBI-C score in our sample for such categorization. However, our aim was not to establish a precise MBI-positive versus MBI-negative dichotomy in our sample of patients but rather to explore the relevance of a high-burden score on other clinical parameters, i.e., to confirm that the presence of NPS as revealed by MBI-C score and cognitive features are not necessarily correlated in AD.^5^ Notwithstanding, further studies should consider a procedure of compilation supervised by qualified personnel.

### Conclusions

In summary, this study provides new evidence that regional network properties could be associated with the presence of NPS in early AD. In particular, the degree centrality (segregation ability) and efficiency (integration ability) of the IFG was negatively associated with mood changes and anxiety. In contrast, a positive correlation was observed between the nodal properties of the PCC and impulse dyscontrol, as well as social inappropriateness, suggesting the presence of potential compensatory mechanisms at play. Overall, these findings underscore the significance of biomarker-based neuroimaging as an effective tool for the characterization of the correlates of NPS in the brain of patients in the AD continuum.

## Supplemental Material

sj-docx-1-alz-10.1177_13872877251316794 - Supplemental material for Structural covariance network patterns linked to neuropsychiatric symptoms in biologically defined Alzheimer's disease: Insights from the mild behavioral impairment checklist Supplemental material, sj-docx-1-alz-10.1177_13872877251316794 for Structural covariance network patterns linked to neuropsychiatric symptoms in biologically defined Alzheimer's disease: Insights from the mild behavioral impairment checklist by Marco Michelutti, Daniele Urso, Benedetta Tafuri, Valentina Gnoni, Alessia Giugno, Chiara Zecca, Maria Teresa Dell’Abate, Davide Vilella, Paolo Manganotti, Roberto De Blasi, Salvatore Nigro and Giancarlo Logroscino in Journal of Alzheimer's Disease
