# Supplementary material for: Structural covariance network patterns linked to neuropsychiatric symptoms in biologically defined Alzheimer's disease: Insights from the mild behavioral impairment checklist
Source: J Alzheimers Dis. 2025 Feb 16;104(2):338–50. doi: 10.1177/13872877251316794 (PMC12231818; doi:10.1177/13872877251316794)
Supplement: sj-docx-1-alz-10.1177_13872877251316794 - Supplemental material for Structural covariance network patterns linked to neuropsychiatric symptoms in biologically defined Alzheimer's disease: Insights from the mild behavioral impairment checklist [file sj-docx-1-alz-10.1177_13872877251316794.docx]

**Supplemental Material**

**Structural covariance network patterns linked to neuropsychiatric symptoms in biologically defined Alzheimer’s disease: Insights from the Mild Behavioral Impairment Checklist**

**Supplemental Table 1.** Scores for MBI-C and neuropsychological assessments

| **Test** | **Mean** | **Standard Deviation** | **Number of subjects** |
| --- | --- | --- | --- |
| MBI-C-total score | 16.00 | 12.43 | 33 |
| MBI-drive/motivation | 4.24 | 3.42 | 33 |
| MBI-Mood/Anxiety | 3.45 | 3.47 | 33 |
| MBI-impulse discontrol | 5.91 | 5.75 | 33 |
| MBI-Social Inappropriateness | 1.39 | 1.84 | 33 |
| MBI-Perception/Thought | 1.00 | 1.80 | 33 |
| RVLT-I_raw | 20.18 | 7.25 | 33 |
| RVLT-I_z-score | -2.17 | 1.03 | 33 |
| RVLT-D_raw | 1.42 | 2.06 | 33 |
| RVLT-D_z-score | -2.27 | 0.82 | 33 |
| RVLT_index_RIC_raw | 0.64 | 0.31 | 30 |
| RVLT_index_RIC_z-score | -2.16 | 1.86 | 30 |
| Rey_Figure_D_raw | 5.22 | 7.79 | 9 |
| Rey_figure_D_z-score | -1.83 | 0.63 | 9 |
| TMT_B_raw | 404.64 | 227.74 | 14 |
| TMT_B_z-score | -1.68 | 2.33 | 14 |
| TMT_B-A_raw | 290.79 | 200.98 | 14 |
| TMT_B-A_z-score | -1.59 | 2.29 | 14 |
| STR_Time_raw | 65.07 | 46.96 | 25 |
| STR_Time_z-score | -2.60 | 4.11 | 25 |
| STR_Errors_raw | 6.98 | 7.07 | 24 |
| STR_Errors_z-score | -1.72 | 2.74 | 25 |
| FAS_raw | 17.09 | 8.45 | 33 |
| FAS_z-score | -1.14 | 0.79 | 33 |
| DS_Inv_raw | 2.42 | 1.12 | 33 |
| DG_I_z-score | -1.66 | 1.11 | 33 |
| Social_Cognition_raw | 7.44 | 3.12 | 25 |
| Social_Cognition_z-score | -1.33 | 1.27 | 25 |
| TMT_A_raw | 127.45 | 74.34 | 29 |
| TMT_A_z-score | -2.03 | 3.03 | 29 |
| SDMT_raw | 17.00 | 13.74 | 6 |
| SDMT_z-score | -2.39 | 0.97 | 6 |
| DS_Dir_raw | 5.18 | 1.07 | 33 |
| DS_Dir_z-score | -0.43 | 1.22 | 33 |
| BOSTON_raw | 11.06 | 3.05 | 33 |
| Boston_z-score | -1.42 | 2.34 | 33 |
| FLUS_raw | 22.03 | 8.77 | 33 |
| FLUS_z-score | -1.53 | 1.08 | 33 |
| FAS_raw | 17.09 | 8.45 | 33 |
| FAS_z-score | -1.14 | 0.79 | 33 |
| CLOCK_raw | 6.76 | 3.48 | 33 |
| Clock_z-score | -1.81 | 1.66 | 33 |
| Rey_Figure_Copy_raw | 22.72 | 12.08 | 9 |
| Rey_Figure_Copy_z_score | -2.20 | 3.33 | 9 |
| AC_raw | 9.84 | 3.19 | 32 |
| AC_z-score | -1.44 | 1.88 | 32 |
| VOSP_L_raw | 14.24 | 5.93 | 29 |
| VOSP_z_score | -2.86 | 4.01 | 29 |
| NPI_tot | 20.41 | 20.19 | 29 |

MBI: mild behavioral impairment; MMSE: Mini-Mental State Examination; FAB: Frontal Assessment Battery; RVLT: Rey Verbal Learning Test; TMT: Trail-Making-Test; STR: Stroop Test; DS_I: Inverse Digit Span; SDMT: Symbol Digit Modality Test; DS_Dir: Direct Digit Span; FLUS: Category Fluencies; AC: Design Copy Test; VOSP: Visual Object and Perception Battery; NPI: Neuropsychiatric Inventory; ADL: Activities of Daily Living; IADL: Instrumental Activity of Daily Living
